# Magnetic Resonance Q Mapping Reveals a Decrease in Microvessel Density in the arcAβ Mouse Model of Cerebral Amyloidosis

**DOI:** 10.3389/fnagi.2015.00241

**Published:** 2016-01-19

**Authors:** Giovanna D. Ielacqua, Felix Schlegel, Martina Füchtemeier, Jael Xandry, Markus Rudin, Jan Klohs

**Affiliations:** ^1^Institute for Biomedical Engineering, ETH and University of ZurichZurich, Switzerland; ^2^Neuroscience Center Zurich, University of Zurich and ETH ZurichZurich, Switzerland; ^3^German Center for Neurodegenerative DiseasesBerlin, Germany; ^4^Department of Experimental Neurology, Charité - University Medicine BerlinBerlin, Germany; ^5^Institute of Pharmacology and Toxicology, University of ZurichZurich, Switzerland

**Keywords:** Alzheimer's disease, microvessel density, dynamic susceptibilty contrast MRI, relaxation rate shift index, superparamagnetic iron oxide nanoparticles, cerebral amyloidosis, cerebral amyloid angiopathy

## Abstract

Alterations in density and morphology of the cerebral microvasculature have been reported to occur in Alzheimer's disease patients and animal models of the disease. In this study we compared magnetic resonance imaging (MRI) techniques for their utility to detect age-dependent changes of the cerebral vasculature in the arcAβ mouse model of cerebral amyloidosis. Dynamic susceptibility contrast (DSC)-MRI was performed by tracking the passage of a superparamagnetic iron oxide nanoparticle in the brain with dynamic gradient echo planar imaging (EPI). From this measurements relative cerebral blood volume [rCBV(DSC)] and relative cerebral blood flow (rCBF) were estimated. For the same animal maps of the relaxation shift index Q were computed from high resolution gradient echo and spin echo data that were acquired before and after superparamagnetic iron oxide (SPIO) nanoparticle injection. *Q*-values were used to derive estimates of microvessel density. The change in the relaxation rates ${\Delta \text{R}}_{2}^{*}$ obtained from pre- and post-contrast gradient echo data was used for the alternative determination of rCBV [rCBV(${\Delta \text{R}}_{2}^{*}$)]. Linear mixed effects modeling found no significant association between rCBV(DSC), rCBV(${\Delta \text{R}}_{2}^{*}$), rCBF, and Q with genotype in 13-month old mice [compared to age-matched non-transgenic littermates (NTLs)] for any of the evaluated brain regions. In 24-month old mice there was a significant association for rCBV(DSC) with genotype in the cerebral cortex, and for rCBV(${\Delta \text{R}}_{2}^{*}$) in the cerebral cortex and cerebellum. For rCBF there was a significant association in the cerebellum but not in other brain regions. *Q*-values in the olfactory bulb, cerebral cortex, striatum, hippocampus, and cerebellum in 24-month old mice were significantly associated with genotype. In those regions *Q*-values were reduced between 11 and 26% in arcAβ mice compared to age-matched NTLs. Vessel staining with CD31 immunohistochemistry confirmed a reduction of microvessel density in the old arcAβ mice. We further demonstrated a region-specific association between parenchymal and vascular deposition of β-amyloid and decreased vascular density, without a correlation with the amount of Aβ deposition. We found that Q mapping was more suitable than the hemodynamic read-outs to detect amyloid-related degeneration of the cerebral microvasculature.

## Introduction

Alzheimer's disease (AD) is a progressive neurodegenerative disorder and the most common form of dementia (Lopez, 2011; Mangialasche et al., 2012). AD pathology is characterized by changes in β-amyloid (Aβ) metabolism, abundance of soluble Aβ oligomers, parenchymal Aβ deposits and neurofibrillary tangles, synaptic dysfunction and neurodegeneration, and loss of cognitive function (Haass and Selkoe, 2007). The pathology is not restricted to the neuronal compartment as it also affects all cell types of the neurovascular unit including circulating leukocytes, endothelial cells, pericytes, perivascular antigen-presenting cells, astrocytes, and microglia (Iadecola, 2004; Grammas, 2011; Zlokovic, 2011). A particular attention has been paid to the vascular system where a number of studies have provided evidence for pronounced cerebrovascular dysfunction in AD.

Cerebral hypoperfusion is an early sign of cerebrovascular dysfunction in AD patients (Hirao et al., 2005; Johnson et al., 2005). Postmortem histological studies have revealed that microvessel density is altered in the AD brain. While some studies reported a reduction in certain brain areas in AD patients (Bell and Ball, 1986; Fischer et al., 1990; Buèe et al., 1997; Bouras et al., 2006), other studies have demonstrated hypervascularity (Desai et al., 2009; Biron et al., 2011). In addition, blood vessels are morphologically abnormal i.e., they become fragmented, twisted, or tortuous with glomerular loop formation (Buèe et al., 1997; Bouras et al., 2006), have an altered vessel wall composition (Scheibel et al., 1987), and impaired structural integrity (Farrall and Wardlaw, 2009). It has been hypothesized that these microvascular distortions can compromise neuronal function and might thus contribute significantly to the cognitive decline in AD (de la Torre, 1994; Ostergaard et al., 2013). Moreover, cerebral amyloid angiopathy that is present in up to 90% of AD patients, involves the deposition of Aβ within the leptomeninges and parenchymal microvessels (Vinters, 1987). Cerebral amyloid angiopathy induces degeneration of smooth muscle cells, impairment of blood-brain barrier function, and the occurrence of cerebral microbleeds, all of which can compromise cognitive function (Cordonnier and van der Flier, 2011).

To date, a variety of transgenic animals that mimic pathological features of AD have been engineered. Among them, transgenic mice overexpressing the mutant amyloid precursor protein (APP) are widely used to model Aβ-related pathologies. APP mice have increased levels of Aβ oligomers, age-dependent deposition of diffuse and fibrillar parenchymal plaques with variable degrees of vascular amyloid formation, deficits in brain metabolism and cognitive function similar to AD patients (Sturchler-Pierrat et al., 1997; Knobloch et al., 2007; Merlini et al., 2011; Kulic et al., 2012). Hypoperfusion and aberrations in microvessel density and morphology have been equally observed in APP mice (Lee et al., 2005; Miao et al., 2005; Kouznetsova et al., 2006; Meyer et al., 2008; Biron et al., 2011; Klohs et al., 2012).

Magnetic resonance imaging (MRI) can be used to non-invasively assess the cerebral vasculature and its function in AD, and has been widely applied to assess vascular dysfunction in APP models (for a recent review see Klohs et al., 2014). For example, magnetic resonance angiography techniques have been used to reveal abnormalities in vessel morphology and density in a variety of APP mouse strains (Beckmann et al., 2003; Thal et al., 2009; El Tayara Nel et al., 2010; Kara et al., 2012; Klohs et al., 2012). Functional MRI techniques have demonstrated a reduction of relative CBV (rCBV), cerebral blood flow (rCBF), and functional connectivity (Wu et al., 2004a; Weidensteiner et al., 2009; Massaad et al., 2010; Faure et al., 2011; Poisnel et al., 2012; Hébert et al., 2013; Zerbi et al., 2013; Grandjean et al., 2014). Moreover, blood-brain barrier impairment and occurrence of cerebral microbleeds were observed with MRI (Beckmann et al., 2011; Klohs et al., 2011, 2013, 2015). The application of these techniques to APP mice is not only useful for the phenotyping of these mouse strains, but combined with histology also enables validation of possibly clinical imaging read-outs.

In this study we aimed to assess age-dependent changes in vascular function in arcAβ mice with different vascular MRI parameters *in vivo*. We intended to compare hemodynamic read-outs related to the overall vasculature such as rCBV and rCBF and to compare those to the relaxation shift index Q, a measure of the microvessel density in the same animal. The arcAβ is an APP overexpressing mouse strain with pronounced vascular deficits and cerebral amyloid angiopathy (Merlini et al., 2011). Dynamic susceptibility contrast (DSC)-MRI measurements were performed by tracking the passage of the SPIO bolus in the brain with dynamic gradient echo (GE) echo planar imaging (EPI) to estimate rCBV(DSC) and rCBF in the same animal (Østergaard, 2005). For Q mapping the change in relaxation rates ΔR_2_ and ${\Delta \text{R}}_{2}^{*}$ were estimated from GE and spin echo (SE) data before and after the injection of superparamagnetic iron oxide (SPIO) nanoparticle injection when the particle concentration reached steady-state (Jensen and Chandra, 2000; Wu et al., 2004b). The change in the relaxation rates ${\Delta \text{R}}_{2}^{*}$ from pre- and post-contrast GE data was used to determine rCBV(${\Delta \text{R}}_{2}^{*}$), for comparison with DSC-MRI. Hemodynamic read-outs and Q maps were compared with immunohistochemical evaluation of microvessel density and Aβ deposition.

## Materials and methods

### Animals

All experimental procedures conformed to the national guidelines of the Swiss Federal Act on Animal Protection and were approved by an official committee (license 194/2011, Cantonal Veterinary Office, Zurich, Switzerland). Male and female transgenic arcAβ mice overexpressing the human APP695 with the Swedish and the Arctic (E693G) mutations (Knobloch et al., 2007) that were backcrossed on a C57Bl/6 background for more than 15 generations and non-transgenic littermates (NTLs) of the same background were used for the study. Batches of animals of 13 ± 1 months of age (arcAβ *n* = 8; NTLs *n* = 8) and 24 ± 1 month of age (arcAβ mice *n* = 8; NTLs *n* = 14) were investigated. Animals were housed in a temperature controlled room in individually ventilated cages, containing up to five animals per cage, under a 12 h dark/light cycle. Food and water were provided *ad libitum*.

### Animal preparation

Anesthesia was induced using 3% isoflurane (Abbott, Cham, Switzerland) in a 4:1 air/oxygen mixture. Mice were endotracheally intubated and mechanically ventilated during measurements with 80 breaths/minute while applying a respiration cycle of 25% inhalation and 75% exhalation (MRI-1 Volume Ventilator, CWI Inc., Ardmore, USA) using 1.5% isoflurane. The tail vein was cannulated for administration of contrast agents. Body temperature was monitored with a rectal temperature probe (MLT415, ADInstruments) and kept at 36.0 ± 0.5°C using a warm-water circuit integrated into the animal support (Bruker BioSpin).

### Physiological monitoring

In a subset of 24-months old animals (*n* = 4) that did not undergo MRI examination the left femoral artery was cannulated to monitor mean arterial blood pressure (MABP) continuously (Transducer and Transbridge Amplifier; World Precision Instruments, Sarasota, USA) and to provide serial measurements of arterial blood gases (Compact 2 AVL, Bad Homburg, Germany).

### Magnetic resonance imaging

Data were acquired on a Bruker BioSpec 94/30 (Bruker BioSpin GmbH) small animal MR system operating at 9.4 T. The system was equipped with a cryogenic 2 × 2 phased-array cryogenic mouse head surface coil (Bruker BioSpin AG, Fällanden, Switzerland).

T_2_-weighted anatomical reference images were acquired using a SE sequence [Rapid Acquisition with Rapid Enhancement (RARE)], with an echo time (TE) = 47 ms, echo spacing = 11.8 ms, repetition time (TR) = 4200 ms, RARE factor = 8. Fifteen sagittal slices of 0.5 mm thickness with a field-of-view = 20 × 20 mm, and a matrix of 384 × 384 were recorded to give a nominal resolution of 52 × 52 μm. In addition, twelve axial images of 0.5 mm thickness were acquired using the balanced Steady-State Free Precession type (TrueFISP) sequence. The imaging parameters were TE = 2.4 ms, TR = 4.8 ms, flip angel α = 60°, excitation pulse length: 1 ms; pixel bandwidth: 586 ms. Data were recorded with a field-of-view = 23.7 × 14 mm and matrix of 256 × 256 yielding an in-plane voxel dimension of 92 × 55 μm.

For Q mapping, global 1st order shimming followed by fieldmap-based local shimming was performed to reduce static magnetic field inhomogeneities. Relaxation rates ΔR_2_and ${\Delta \text{R}}_{2}^{*}$ were acquired before and after SPIO injection using a SE and GE sequence, respectively. 2D SE images were acquired with TE = 30 ms, TR = 2000 ms, RARE factor = 8, in coronal direction, with 6 averages. Three slices of 0.6 mm thickness and a 0.6 mm slice gap were acquired with a field-of-view = 19.2 × 19.2 mm, and a matrix of 192 × 192 to give a nominal resolution of 100 × 100 μm. The 3D GE sequences (FLASH) were applied with the parameters TE/TR = 5.5/40 ms, α = 5°, bandwidth of 50 kHz and eight averages. A slab with field-of-view = 19.2 × 19.2 × 4 mm, and a matrix of 192 × 192 × 40 was recorded to give a spatial resolution of 100 × 100 × 100 μm. The three SE images were aligned within the field-of-view of the GE image.

Between pre- and postcontrast images, DSC-MRI data was acquired with GE EPI with TE = 10 ms, TR = 400 ms, α = 90°, bandwidth = 238 kHz, and no averaging. Twelve 0.5 mm thick slices with a slice gap of 0.5 mm were recorded in axial orientation with a field-of-view = 23.7 × 23.7 mm, and a matrix of 64 × 64 to give a nominal resolution of 370 × 219 μm. A series of 300 images with a temporal resolution of 400 ms were acquired. Animals were injected intravenously with a bolus of SPIOs (30 mg Fe/kg body weight, Endorem, Guerbet) with an infusion pump at a constant flow of 2 ml/min (Harvard apparatus) after 30 s of recorded baseline. The total procedures lasted about 2.5–3 h per animal.

### Data processing

From the dynamic GE EPI sequence, rCBV(DSC), and rCBF maps were generated by voxel-wise fitting the DSC-MRI data using custom-written MATLAB code (The MathWorks, Natick, MA) based on a previously described algorithm (Kim et al., 2010). Signal-time curves were converted to the transverse relaxation rate change which reflects concentration-time relationship with the following equation (Østergaard, 2005):

(1) $$\begin{array}{l} C_{t}\left(t\right)\propto \Delta R_{2}\left(t\right)\;=\;-\frac{k}{TE}\ln\;\left(\frac{S\left(t\right)}{S_{0}}\right) \end{array}$$

where *C*_*t*_(*t*) is the contrast agent concentration in tissue at time *t*, ΔR_2_(*t*) is the relaxation rate of the voxel at time *t*; *S*(*t*) is the signal intensity of the voxel at time *t*, *S*_0_ is the precontrast signal intensity, *k* is the proportionality factor, and *TE* is the echo time (ms).

After brain masking, the contrast bolus arrival time was estimated using a linear-quadratic piecewise continuous regression model (Cheong et al., 2003). A gamma-variate curve was then fitted to the relevant part [from bolus arrival time to bolus arrival time + 2.5 × (bolus arrival time-time to peak)] of the concentration-time curve by a least squares fit (Madsen, 1992). The arterial input function was estimated from a search algorithm using an artery-likelihood metric. The rCBV(DSC) was then computed as the integral of the fitted gamma-variate curve, normalized over the integral of the arterial input function:

(2) $$\begin{array}{l} rCBV=\frac{\overset{\infty }{\underset{-\infty }{\int }}C_{t}\left(\tau \right)d\tau }{\overset{\infty }{\underset{-\infty }{\int }}C_{a}\left(\tau \right)d\tau } \end{array}$$

rCBF was estimated by deconvolving the measured concentration time curve *C*_*t*_(τ) with the change in the feeding artery *C*_*a*_(*t*). Deconvolution was achieved using singular value decomposition with a truncation parameter r_trunc_ = 0.2.

From the pre- and postcontrast SE and GE images the transverse relaxation rates were computed according to:

(3) $$\begin{array}{l} \Delta R_{2}=\frac{1}{TE}\ln\;\left(\frac{S_{pre}}{S_{post}}\right) \end{array}$$

(4) $$\begin{array}{l} \Delta R_{2}^{*}=\frac{1}{TE}\ln(\frac{S_{pre}^{*})}{S_{post}^{*})} \end{array}$$

where *S*_*pre*_ and *S*_*post*_ represent the precontrast and postcontrast signal intensities for the SE images and *S*${}_{pre}^{*}$ and *S*${}_{post}^{*}$ the precontrast and postcontrast signal intensities for the GE images, and TE is the echo time.

Maps of the relaxation shift index *Q* where computed voxel-wise according to Wu et al. (2004b):

(5) $$\begin{array}{l} Q\equiv \frac{\Delta R_{2}}{{\left(\Delta R_{2}^{*}\right)}^{2∕3}} \end{array}$$

The ${\Delta \text{R}}_{2}^{*}$ images were resampled on the grid of the ΔR_2_ images using cubic spline interpolation to achieve the same spatial resolution. And all negative values for ΔR_2_ and ${\Delta \text{R}}_{2}^{*}$ were set to zero to avoid complex values. Microvessel density N was calculated according to Wu et al. (2004b):

(6) $$\begin{array}{l} N\approx Q^{3}\times 329s∕{mm}^{2} \end{array}$$

For a steady-state blood tracer concentration to the total amount of tracer in the voxel is proportional to local rCBV and thus ${\Delta \text{R}}_{2}^{*}$ yields estimation of rCBV (Berry et al., 1996). We have denoted this as rCBV(${\Delta \text{R}}_{2}^{*}$).

One 13-month old arcAβ mouse was excluded from the analysis because of movements between pre and post-contrast agent scan.

### Data analysis

Regions-of-interest (ROIs) were drawn manually on coronal pre-contrast 2D SE images by a person blinded to the experimental groups and then transferred onto Q and ${\Delta \text{R}}_{2}^{*}$ maps. The olfactory bulb, cerebral cortex, striatum, hippocampus, thalamus, and cerebellum were identified according to an anatomical mouse brain atlas (Franklin and Paxinos, 1997). In addition, ROIs over the cerebral cortex, hippocampus, and cerebellum were drawn manually on the TrueFISP images and transferred onto rCBF and rCBV(DSC) maps using MATLAB based software Aedes (http://aedes.uef.fi).

### Immunohistology

After MRI measurements mice were decapitated and brains were removed and snap frozen in 2-methylbutane (Sigma-Aldrich), precooled to −60°C. Brains were cut into 10 μm coronal sections with a cryostat (Thermo Fisher CryoStar NX70). After air-drying, sections were fixed in acetone at room temperature and placed in Bond wash solution. Sections were incubated with the primary antibodies anti-murine CD31 (Rat, 1:250, catalog#NB100-1642; Novus Biological) and anti-β-amyloid (Mouse, 1:3000 Aβ1-16 specific, catalog#SIG 3920, Signet Covance) for 45 min in a humid chamber. After two washing steps in Bond wash solution for 5 min each, sections were incubated for 45 min at room temperature in humid chamber with secondary antibodies: anti-rat DyLight 549 (Donkey, 1:200, catalog#712-506-153, Jackson Immunoresearch) and anti-mouse DyLight 406 (Donkey, 1:200, catalog#715-475-151, Jackson Immunoresearch). After the second incubation, sections were washed in Bond wash solution and embedded with aqueous medium (Medi-Mount, Medite, Germany). Sections were scanned with a Pannoramic SCAN digital slide scanner (3DHISTECH, Hungary). CD31 and Aβ were excited and detected with DAPI and Alexa 568 filters (λex = BP387/11, λem = BP 440/40 and λex = BP560/25 m, λem = BP 607/36 respectively). Vascular density was qualitatively evaluated in the same ROIs investigated with Q mapping using random fields of view (size = 862 × 1330 μm). Aβ deposits were clearly visible in arcAβ mice as parenchymal plaques and vascular deposits with no specific fluorescence in sections of NTLs (**Figures 5**, **6**).

### Statistical analysis

Data are presented as box plots (http://boxplot.tyerslab.com/). For each brain region, linear mixed effects analysis was performed using R (https://www.r-project.org/) and lme4 (Bates et al., 2015). Genotype and age were considered as fixed effects, while individual animals were entered as random effects into the model. Values of *P* < 0.05 were considered significant.

## Results

### Physiological parameters during data acquisition

Since data acquisition for Q mapping requires long scan times during which mice need to be anesthetized, we monitored animal physiology over a 2 h period of isoflurane anesthesia in 24-month old NTLs and arcAβ (Supplementary Table 1). In both groups arterial blood pressure and blood gas values did not differ between NTLs and arcAβ mice.

### Assessing vascular parameters with MRI

**Figure 1:**
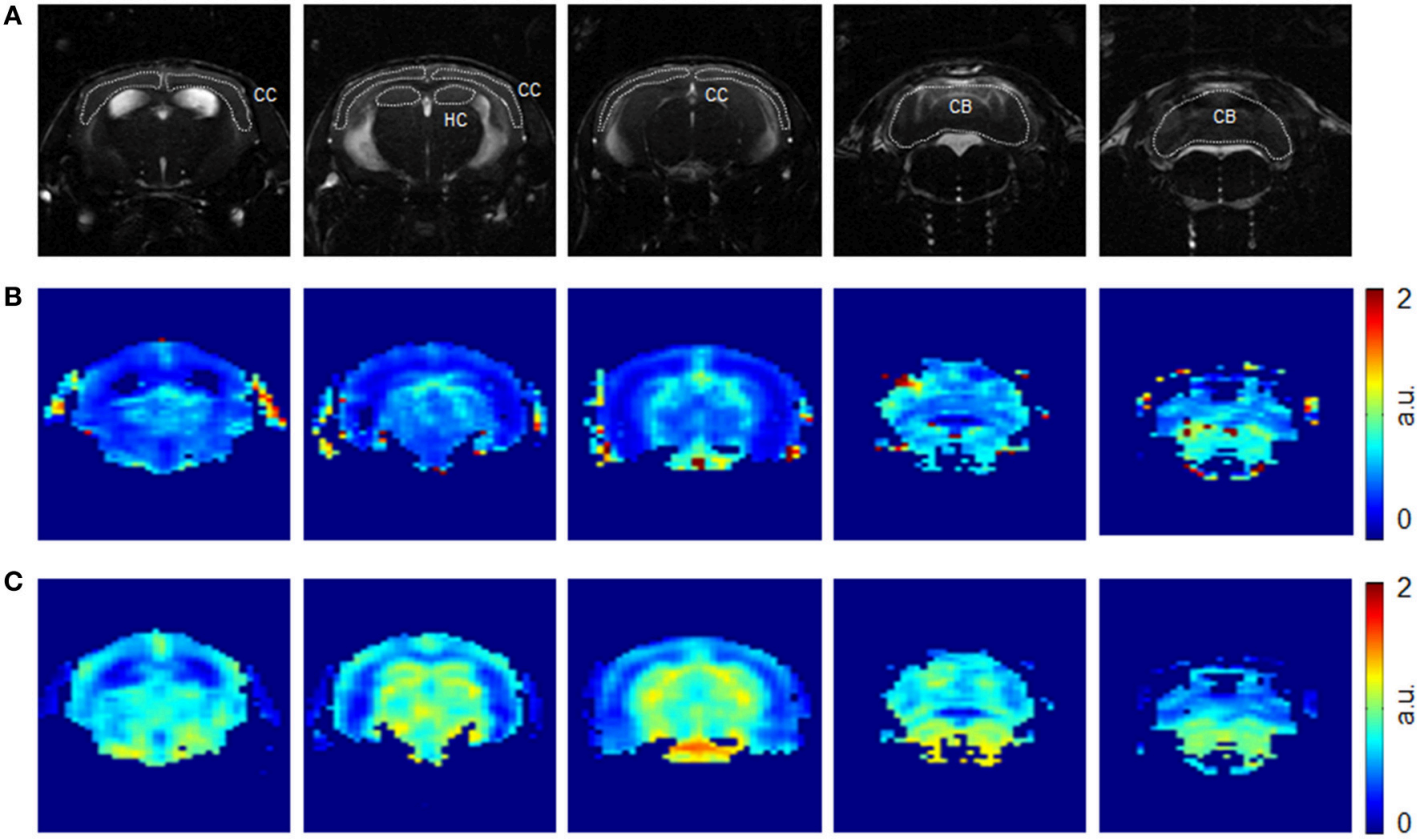
**Representative axial images of a mouse brain at different anatomical locations**. Images were acquired with a TrueFISP sequence **(A)** before injection of SPIOs. Corresponding rCBV(DSC) **(B)** and rCBF **(C)** maps obtained from the dynamic GE EPI sequence. Region-of-interests were drawn over the cerebral cortex (CC), hippocampus (HC), and cerebellum (CB) as indicated.

**Figure 2:**
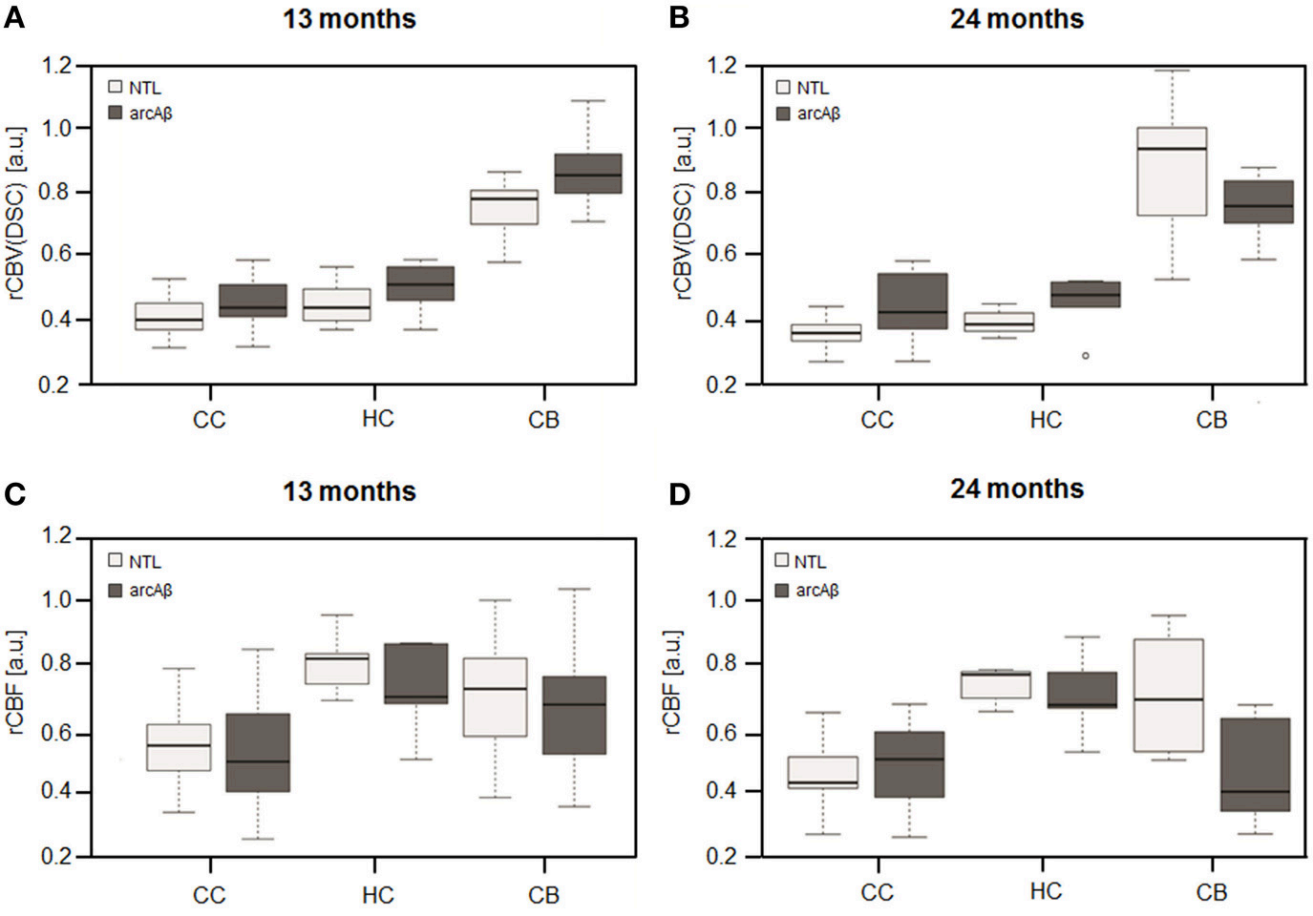
**Box plots of rCBV(DSC) (A,B) and rCBF (C,D) from DSC-MRI**. Values were measured in the cerebral cortex (CC), hippocampus (HC), and cerebellum (CB) in 13-months **(A,C)** and 24-months **(B,D)** old arcAβ mice and non-transgenic littermates (NTL). Center lines show the medians; box limits indicate the 25th and 75th percentiles; whiskers extend 1.5 times the interquartile range from the 25th and 75th percentiles, outliers are represented by dots.

Representative rCBV(DSC) and rCBF maps of mice brains are depicted in Figures 1A–C. In different brain regions rCBV(DSC) and rCBF values were measured by ROI analysis. The corresponding box plots are depicted in Figures 2A–D. Linear mixed effect modeling found no significant association between rCBV(DSC) or rCBF and genotype in 13-month old mice for any of the evaluated brain regions. In 24-month old mice there was a significant association for rCBV(DSC) with genotype in the cerebral cortex, while there was no association in the hippocampus and cerebellum (Table 1). The rCBV in the cerebral cortex was increased by 21%. In contrast, for rCBF there was a significant association with genotype in the cerebellum of 24-month old mice, but no association in the cerebral cortex and hippocampus. The rCBF was reduced by 35% in the cerebellum of the arcAβ compared to non-transgenic controls.

**Table 1:** Linear mixed effect analysis of rCBV(DSC), rCBV(${\Delta \text{R}}_{2}^{*}$) rCBF, and *Q*-values measured in different brain regions.

	**13-months old**	**24-months old**
**rCBV(DSC)**		
Cerebral cortex	0.470	**0.007**
Hippocampus	0.813	0.262
Cerebellum	0.086	0.106
**rCBF**		
Cerebral cortex	0.334	0.208
Hippocampus	0.138	0.656
Cerebellum	0.282	<**0.001**
**rCBV(ΔR**${}_{2}^{*}$)		
Olfactory bulb	0.902	0.996
Cerebral cortex	0.890	**0.002**
Striatum	0.117	0.197
Hippocampus	0.684	0.078
Thalamus	0.629	0.173
Cerebellum	0.088	**0.018**
**Q**		
Olfactory bulb	0.590	**0.026**
Cerebral cortex	0.667	**0.001**
Striatum	0.766	**0.001**
Hippocampus	0.949	**0.003**
Thalamus	0.911	0.397
Cerebellum	0.516	**0.004**

*Shown are the p-values for the genotype effects for each age group. Highlighted are statistically significant effects*.

**Figure 3:**
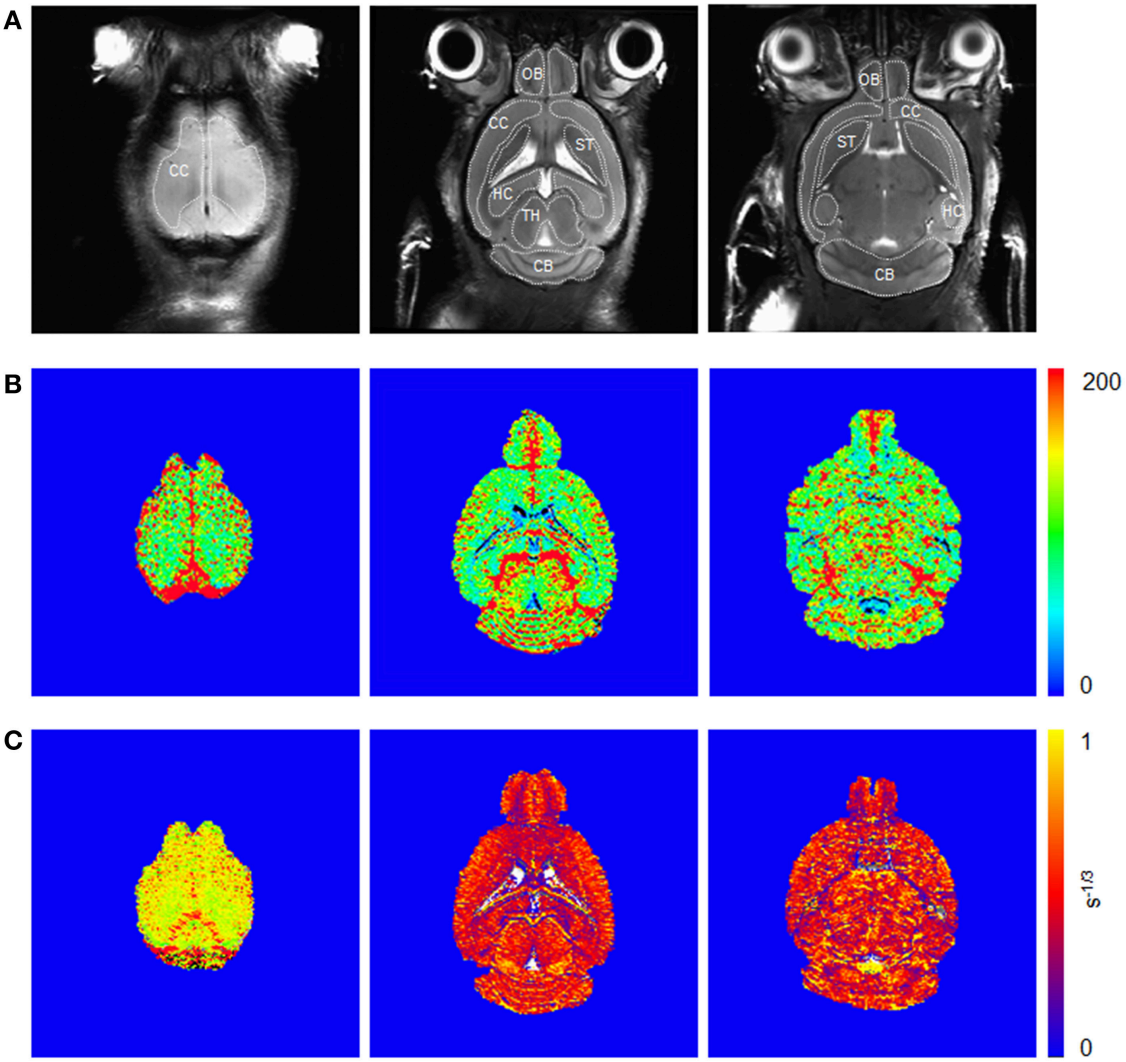
Coronal spin echo images (A) before injection of SPIOs and corresponding ${\Delta \text{R}}_{2}^{*}$ (B) and Q maps (C) from steady-state measurements. Illustration of region-of-interests are delineated for the olfactory bulb (OB), cerebral cortex (CC), striatum (ST), hippocampus (HC), thalamus (TH), and cerebellum (CB).

**Figure 4:**
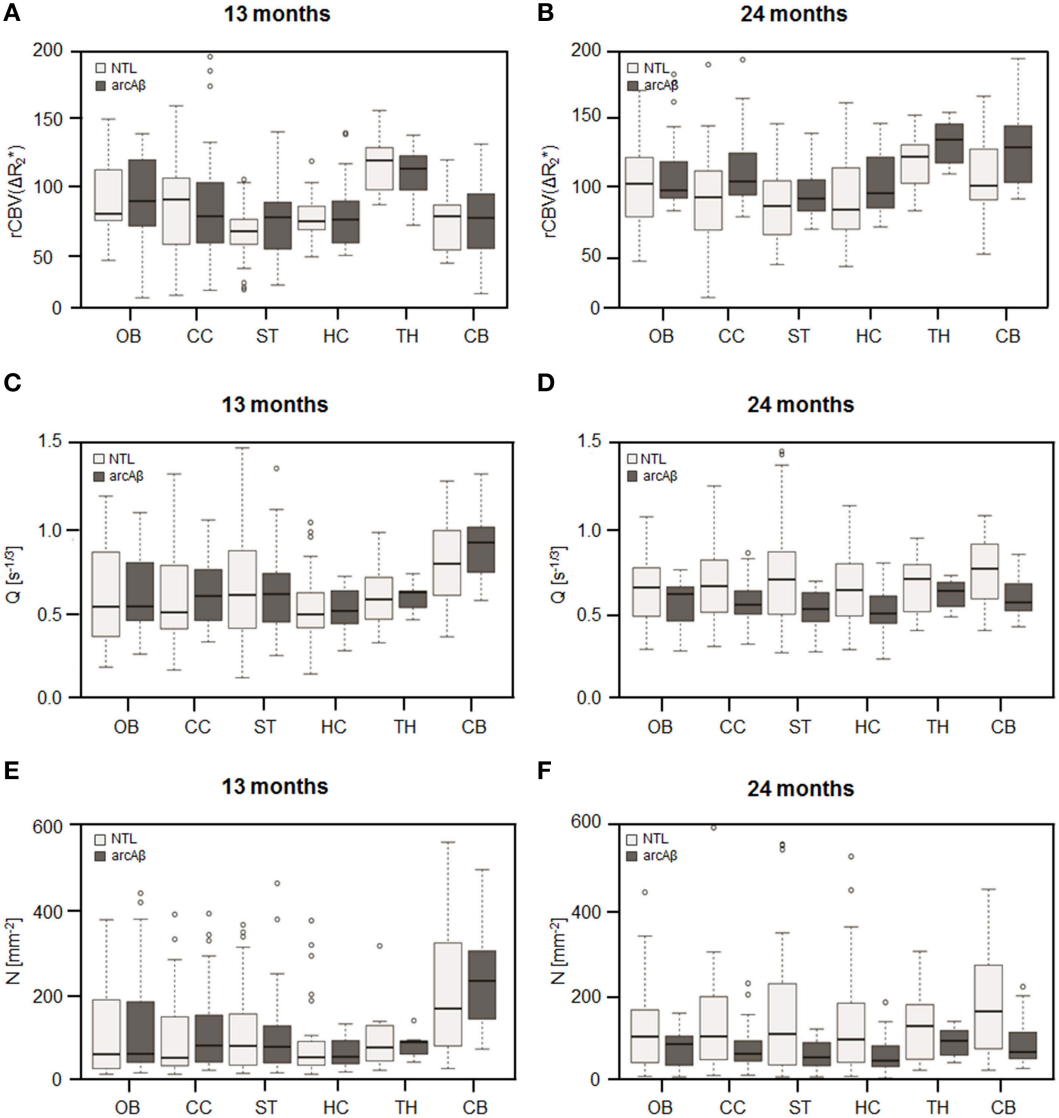
Box plots of the rCBV(${\Delta \text{R}}_{2}^{*}$) (A,B), relaxation shift index Q (C,D), and microvessel density N (E,F) in the olfactory bulb (OB), cerebral cortex (CC), striatum (ST), hippocampus (HC), thalamus (TH), and cerebellum (CB) in 13-months (A,C,E) and 24-months (B,D,F) old arcAβ mice and non-transgenic littermates (NTL). Center lines show the medians; box limits indicate the 25th and 75th percentiles; whiskers extend 1.5 times the interquartile range from the 25th and 75th percentiles, outliers are represented by dots.

Examples of high resolution spin echo images, rCBV(${\Delta \text{R}}_{2}^{*}$) and Q maps are shown in Figure 3. ROI analysis obtained values for rCBV(${\Delta \text{R}}_{2}^{*}$) and Q as shown in box plots in Figures 4A–D. The vessel density N was calculated from *Q*-values for each brain region (Figures 4E,F). Linear mixed effect modeling found no significant association between ${\Delta \text{R}}_{2}^{*}$ and genotype in 13-month old mice for any of the evaluated brain regions. In 24-month old mice there was a significant association for rCBV(${\Delta \text{R}}_{2}^{*}$) with genotype in the cerebral cortex and cerebellum, while there was no association in the other brain regions (Table 1). The rCBV(${\Delta \text{R}}_{2}^{*}$) in the cerebral cortex and in the cerebellum were both increased by 25%. There was no significant association for *Q*-values with genotype in 13-month old mice in all regions tested. *Q*-values in the olfactory bulb, cerebral cortex, striatum, hippocampus, and cerebellum in 24-month old mice were significantly associated with genotype, while there was no statistical association for the thalamus. In 24-month old arcAβ mice *Q*-values were reduced by 11% in the olfactory bulb, 15% in the cerebral cortex, 26% in the striatum, 20% in the hippocampus, and 19% in the cerebellum compared to age-matched NTLs.

### Assessment of microvessel density with vessel staining

**Figure 5:**
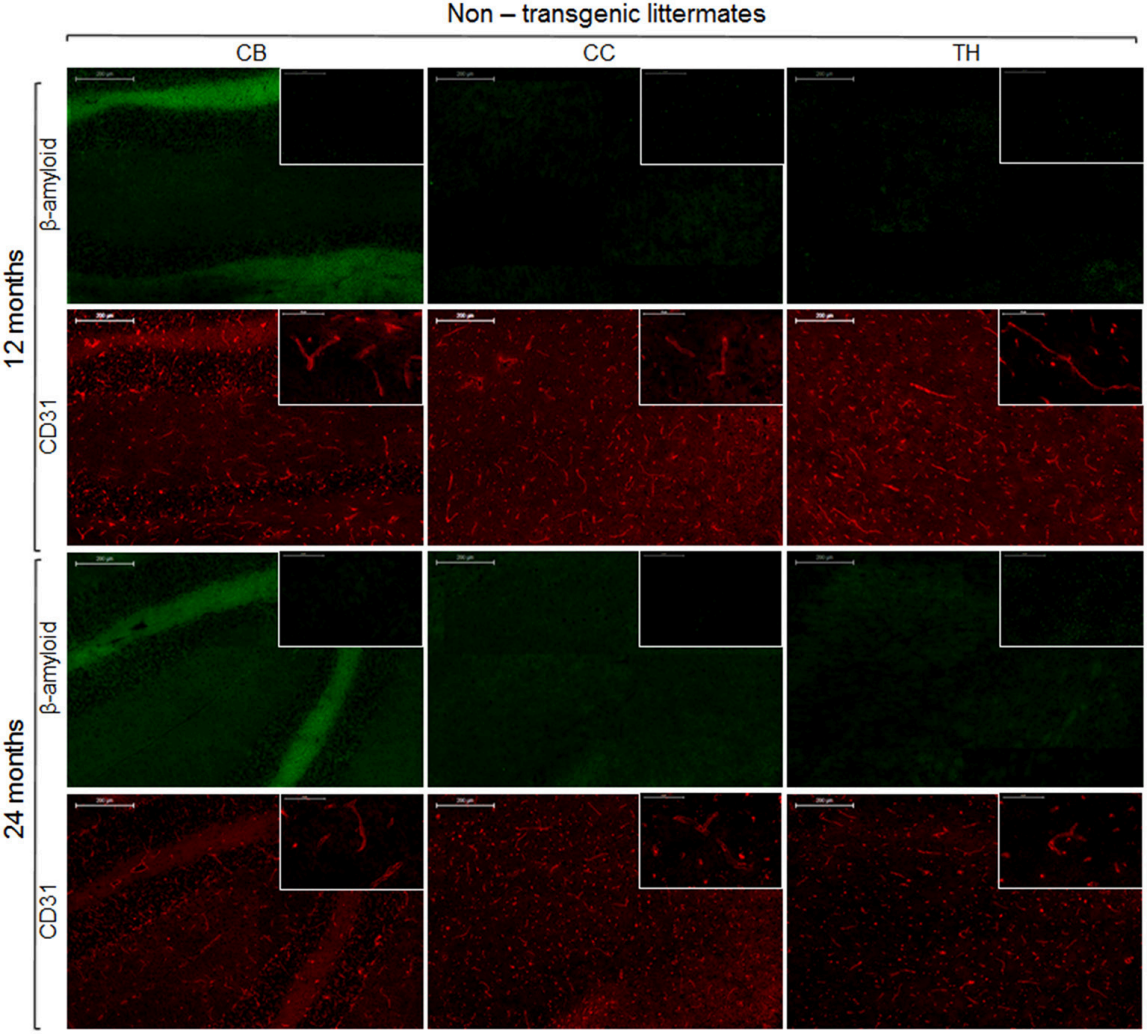
**Immunohistochemistry for vessel staining (CD31) and β-amyloid comparing 13- and 24-month old non-transgenic littermates**. Overviews (scale bars = 200 μm) of the corpus callosum (CC), thalamus (TH), and cerebellum (CB). Insets show the regions with higher magnification (scale bar = 50 μm).

**Figure 6:**
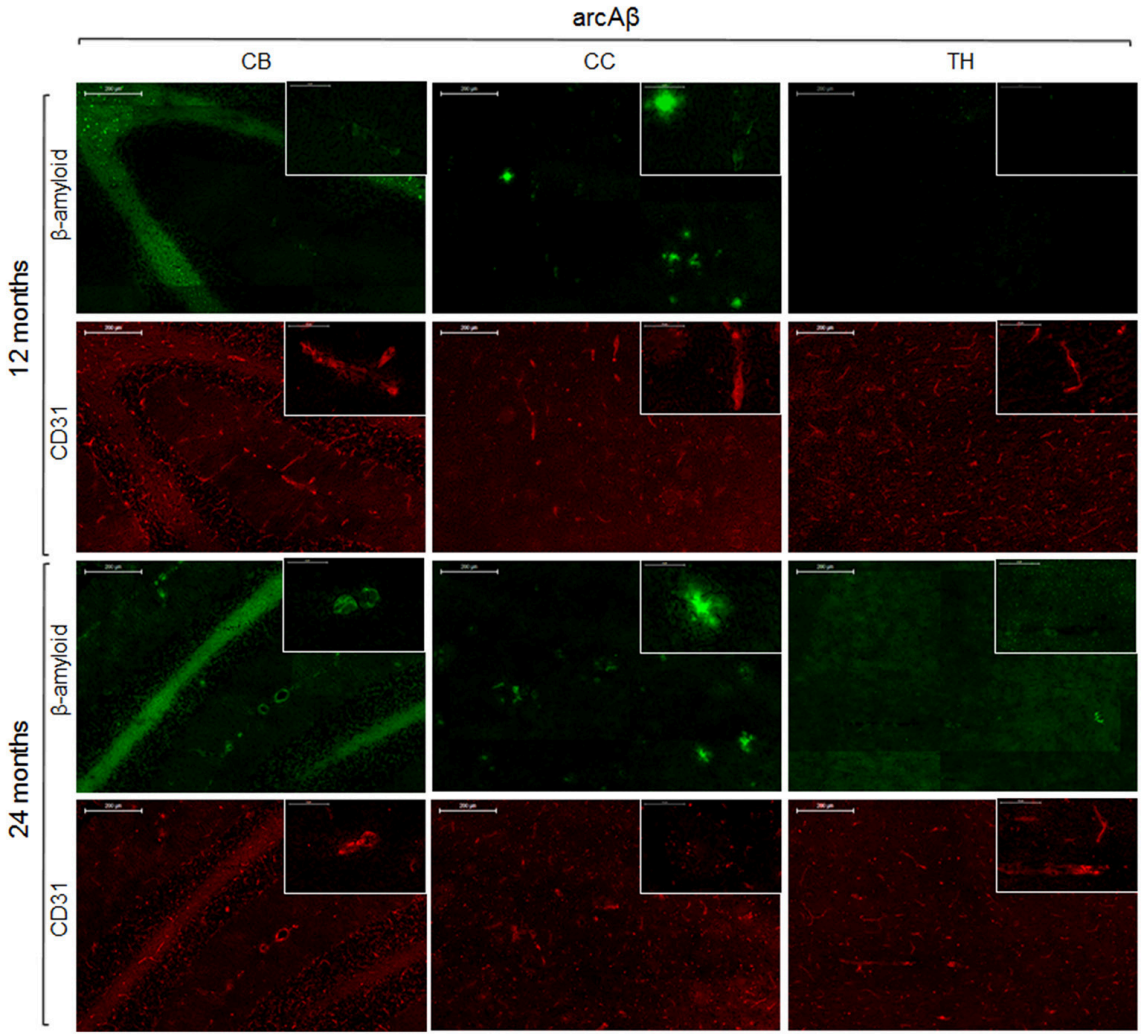
**Immunohistochemistry for vessel staining (CD31) and β-amyloid comparing 13- and 24-month old arcAβ mice**. Overviews (scale bars = 200 μm) of the corpus callosum (CC), thalamus (TH), and cerebellum (CB). Insets show the regions with higher magnification (scale bar = 50 μm).

To reveal the density of cerebral blood vessels, coronal cryosections were immunostained for CD31. Representative images of vessel stainings for the normal mouse brain of different ages are presented in Figure 5. In the investigated brain regions, there were no region-specific differences in immunoreactivity, and there were no differences in vessel densities between 13- and 24-month old NTLs. Figure 6 depicts examples of anti-CD31 immunostaining for arcAβ mice of different ages. Vessel densities were reduced in all brain regions examined, except for the thalamus. Vessel density reduction was most apparent in the cerebral cortex, and cerebellum of 24-month old arcAβ mice compared to age-matched NTLs, while there was no apparent difference in vessel density in the thalamus. Anti-Aβ immunohistochemistry revealed the presence of vascular and parenchymal Aβ deposits in all brain regions examined with exception of the thalamus in 13- and 24-month old arcAβ mice, with an age-dependent increase in the number of fibrillar plaques (Figure 6). In the cerebellum of 24-month old arcAβ mice mainly vascular Aβ deposits were observed with few parenchymal Aβ deposits. NTLs had no Aβ immunoreactivity (Figure 5).

## Discussion

In the present work we applied a protocol that yielded the four MRI parameters rCBV(DSC), rCBV(${\Delta \text{R}}_{2}^{*}$) and, rCBF and the relaxation shift index Q, all describing function of the cerebral vasculature, in the same animal. For this purpose we have combined methods that use iron oxide contrast agent administration. Moreover, the protocol enabled us to compare directly rCBV estimates from DSC-MRI and steady-state measurement of ${\Delta \text{R}}_{2}^{*}$. We investigated which of the read-outs is most suitable to detect pathological changes of the cerebral microvasculature induced by amyloidosis in transgenic arcAβ mice.

Estimates of rCBV from DSC-MRI showed a smaller variance compared to values derived from steady-state measurements of ${\Delta \text{R}}_{2}^{*}$. We observed no difference in rCBV estimates in 13-month old animals. In 24-month old arcAβ mice a significant increase in rCBV(DSC) and rCBV(${\Delta \text{R}}_{2}^{*}$) were found in the cerebral cortex. Only for the cerebellum the analysis yielded differences for the two methods as we found no differences in rCBV(DSC) but a significant increase in rCBV(${\Delta \text{R}}_{2}^{*}$) values. Our finding are in contrast to previous studies which have estimated rCBV in different APP mouse strains by measuring ΔR_2_ or ${\Delta \text{R}}_{2}^{*}$ after injection of an iron oxide contrast agent (Wu et al., 2004a; Weidensteiner et al., 2009). Wu et al. (2004a) found a decrease in rCBV in the cerebral cortex, hippocampus and thalamus, in 4-month old PDAPP mice compared to non-transgenic controls. Weidensteiner et al. (2009) reported reduced rCBV values for the cortex and hippocampus in 17-month old B6.Ps2APP mice and a reduction in rCBF in the occipital cortex of 10- to 17-month-old mice compared to control.

Moreover, we additionally obtained rCBF values with the DSC-MRI. We observed a decrease in rCBF in the cerebellum, and no differences in the other brain regions. This is in contrast to other studies that have assessed cerebral perfusion in transgenic APP mice by applying arterial spin labeling techniques (Massaad et al., 2010; Faure et al., 2011; Poisnel et al., 2012; Hébert et al., 2013). Reductions in rCBF was found in the cortex in 6-month-old APPxPS1-Ki (Faure et al., 2011), 12-month old APP/PS1 (Poisnel et al., 2012), 3-month-old J20 hAPP (Hébert et al., 2013), and in 12- to 16-month-old Tg2576 mice (Massaad et al., 2010).

With Q mapping we found average values of 0.63 ± 0.11 s^−1∕3^ for gray mater regions in 13-month old NTLs. This is in good agreement with *Q*-values of 0.72 ± 0.21 s^−1∕3^ that were estimated for similar regions by Jensen and Chandra (2000), but is lower compared to *Q*-values of 0.95 ± 0.05 s^−1∕3^ reported by Wu et al. (2004b). Differences in *Q*-values might be attributed to differences in mouse strains and ages, but also in the drawing of the ROIs which might lead to the inclusions of different proportions of larger vessels within a ROI (Wu et al., 2004a). Weidensteiner et al. (2009) have previously applied Q mapping to assess microvessel density in B6.Ps2APP mice. They found no difference in vessel density in the brains of 17-month old B6.Ps2APP mice, despite the fact that an immunohistological study had shown a reduction in microvessel density in the cortex in the same mouse strain and age (Kouznetsova et al., 2006). We measured decreased microvessel density in different brain regions in 24-month old arcAβ mice, which were corroborated by results of vessel staining using anti-CD31 immunohistochemistry. A reduction in microvessel density was shown to occur in brain areas that were previously shown to be predominant sites of Aβ deposition (Knobloch et al., 2007; Klohs et al., 2013), and which was confirmed in this study using CD31 vessel staining. The decrease in microvessel density is clearly age-dependent as there was no reduction in 13-month old arcAβ mice.

Differences in hemodynamic read-outs might arise from the different transgenic APP mouse strains used in the studies with differences in onset and severity of amyloid pathology, and a different degree of vascular involvement (Klohs et al., 2014). However, the rCBV, and, in a more complicated relation, the rCBF are both proportional to the fraction of vessels within the voxel (Pathak et al., 2001), and we expected to observe a decrease in rCBV and rCBF in areas with reduced microvessel density. However, in most cases rCBV (both DSC and ${\Delta \text{R}}_{2}^{*}$) and rCBF were unchanged or even increased in 24-month old arcAβ, indicating an unaffected or increased blood vessel density and/or size in the brain regions. That we did not find an association between rCBV, rCBF and microvessel density might be likely explained by the constraints of the methods to derive hemodynamic parameters. It has been previously shown that Aβ pathology in the arcAβ mouse strain affects mainly the microvasculature (Merlini et al., 2011; Klohs et al., 2012, 2015) and this goes together with the reduction in the density of microvessels that we observed in this study. However, both DSC-MRI and steady-state measurements of rCBV using an intravascular iron oxide contrast agent are inherently sensitive to all vessels within a voxel (Simonsen et al., 2000; Zerbi et al., 2013) and it might be conceivable that focal reductions in rCBV and rCBF might be concealed in larger voxels by larger vessels that are not affected by disease. Compared with these methods, Q mapping has been shown to be sensitive to the density of the microvasculature (Jensen and Chandra, 2000) and has thus revealed the decrease in microvessel density that we have also found with immunohistochemistry.

With CD31 vessel staining we observed a clear decrease in cerebral microvessel density in 24-month old arcAβ in brain regions that also showed a reduction in Q and N. The decreased immunoreactivity in these brains suggests that there is an extensive loss of endothelial cells in the advanced disease stage. The present findings are highly consistent with numerous neuropathological reports describing a degeneration of microvessels in certain brain regions in AD patients (Bell and Ball, 1986; Fischer et al., 1990; Buèe et al., 1997; Bouras et al., 2006) and APP mice (Lee et al., 2005; Kouznetsova et al., 2006). Differences exist with respect to the cerebellum where we found a decreased microvessel density in the arcAβ mouse. Previous studies (Knobloch et al., 2007; Klohs et al., 2013) and histological analysis have shown that the cerebellum in this strain is affected by, though mainly vascular, Aβ deposition.

Many studies have described the pathogenic effect of Aβ on the vasculature. For example, neuropathological studies demonstrated that Aβ is associated with structural changes in the vessels including loss of smooth muscle cells, fibrinoid necrosis, and weakening of the vessel wall (Greenberg et al., 1995) as well as apoptosis (Miao et al., 2005). *In vitro* studies have demonstrated that fibrillar Aβ, but not soluble Aβ oligomers, promotes cell degeneration and apoptosis in primary cultures of cerebrovascular smooth muscle cells and pericytes (Van Nostrand et al., 1997; Verbeek et al., 1997; Davis et al., 1999). Additionally, in culture Aβ has been shown to be toxic to endothelial cells (Thomas et al., 1996; Price et al., 2001). This is in line with our finding that the thalamus which was devoid of Aβ deposits had a normal vessel density. However, there were no correlation between the extent of Aβ deposits and the decrease in microvessel density as the striatum which has shown the largest decrease in Q has a rather low number of Aβ deposits compared to the cerebral cortex which has a high Aβ load but a smaller reduction in Q. Moreover, a reduction of microvessel density was observed in the cerebellum where mainly vascular Aβ was observed, indicating that parenchymal Aβ is not required for the degeneration of the microvessels.

To date, the interaction between pathological alterations of the cerebral microvasculature, changes in hemodynamic and cognitive function in AD pathogenesis is not well understood. Using neuroimaging cerebral hypoperfusion has been shown to occur in AD patients and already in patients with mild cognitive impairment (Hirao et al., 2005; Johnson et al., 2005). Histological studies have revealed a reduction in microvessel density in AD patients with dementia (Bell and Ball, 1986; Fischer et al., 1990; Buèe et al., 1997; Bouras et al., 2006). As such studies rely on the examination of postmortem samples that are become largely available during the late stage of the disease it remains unclear at which stage the microvasculature starts to degenerate and how this is related to changes in hemodynamics. Moreover, it is conceivable that a reduction in microvessel density will affect tissue viability, but future studies are warranted to assess the relationship between the extent of microvascular degeneration and the degree of cognitive impairment. The application of Q mapping to patients with brain tumor (Donahue et al., 2000; Schmainda et al., 2004) and cerebral ischemia (Xu et al., 2011, 2012) has been already demonstrated. Previous reports have shown a good correlation between histological and Q mapping measures of microvessel density (Ullrich et al., 2011; Lemasson et al., 2013). In this study we have found Q mapping more suitable than the assessment of hemodynamic parameters, suggesting that Q mapping could be employed to non-invasively and quantitatively assess degeneration of the microvasculature in AD patients. Assessing microvessel density could thus provide a useful tool for the characterization and clinical staging of vascular dysfunction in patients with AD and cerebrovascular diseases.

## Author contributions

Conceived and designed the experiments: JK. Acquired data: GI, MF, JX. Analyzed and interpreted the data: GI, FS, MF, JX, MR, JK. Wrote the manuscript: GI, JK. Made critical revisions to the manuscript: FS, MF, MR.

## Funding

The work was founded by the Swiss National Science Foundation (Grant PZ00P3_136822 to JK) and the EMDO foundation (to JK).

### Conflict of interest statement

The authors declare that the research was conducted in the absence of any commercial or financial relationships that could be construed as a potential conflict of interest.
