# Supplementary material for: Magnetic Resonance Q Mapping Reveals a Decrease in Microvessel Density in the arcAβ Mouse Model of Cerebral Amyloidosis
Source: Front Aging Neurosci. 2016 Jan 19;7:241. doi: 10.3389/fnagi.2015.00241 (PMC4717293; doi:10.3389/fnagi.2015.00241)
Supplement: Supplementary file 1 [file Table1.DOCX]

Supplementary Material

**Decreased microvessel density in the arcAβ mouse model of cerebral amyloidosis revealed by 9.4 T magnetic resonance Q mapping**

**Giovanna D. Ielacqua, Felix Schlegel, Martina Füchtemeier, Jael Xandry, Markus Rudin, Jan Klohs**

**Correspondence:** Jan Klohs, klohs@biomed.ee.ethz.ch

# Supplementary Table

**Physiological parameter in 24-month old NTL and arcAβ mice during 2h of isoflurane anesthesia**

|  | **baseline** | | **1h 15 min** | | **2h** | |
| --- | --- | --- | --- | --- | --- | --- |
|  | **NTL** | **arcAβ** | **NTL** | **arcAβ** | **NTL** | **arcAβ** |
| **MABP (mmHg)** |  |  |  |  |  |  |
| **pCO_2_ (mm Hg)** | 34±6 | 31±2 | 30±3 | 28±1 | 21±9 | 33±0 |
| **pO_2_ (mm Hg)** | 153±51 | 156±9 | 131±69 | 167±27 | 117±49 | 156±13 |
| **pH** | 7.27 | 7.33±0.09 | 7.38±0.10 | 7.43±0.04 | 7.45±0.19 | 7.4±0.04 |

Data are presented as mean ± SD (*n* = 2). MABP, mean arterial blood pressure.
